# Carbohydrate Accumulation and Differential Transcript Expression in Winter Wheat Lines with Different Levels of Snow Mold and Freezing Tolerance after Cold Treatment

**DOI:** 10.3390/plants9111416

**Published:** 2020-10-23

**Authors:** Erika B. Kruse, Samuel Revolinski, Jesse Aplin, Daniel Z. Skinner, Timothy D. Murray, Charles G. Edwards, Arron H. Carter

**Affiliations:** 1 Department of Crop and Soil Science, Washington State University, Pullman, WA 99164, USA; erika.kruse@wsu.edu (E.B.K.); samuel.revolinski@wsu.edu (S.R.); 2 School of Food Science, Washington State University, Pullman, WA 99164, USA; jesse.aplin@wsu.edu (J.A.); edwardsc@wsu.edu (C.G.E.); 3 Wheat Health, Genetics and Quality Research Unit, USDA-ARS, Pullman, WA 99164, USA; dzs@wsu.edu; 4 Department of Plant Pathology, Washington State University, Pullman, WA 99164, USA; tim.murray@wsu.edu

**Keywords:** *Triticum aestivum*, snow mold tolerance, freezing tolerance, carbohydrate accumulation, transcript expression, functional enrichment analysis

## Abstract

Winter wheat (*Triticum aestivum* L.) undergoes a period of cold acclimation in order to survive the ensuing winter, which can bring freezing temperatures and snow mold infection. Tolerance of these stresses is conferred in part by accumulation of carbohydrates in the crown region. This study investigates the contributions of carbohydrate accumulation during a cold treatment among wheat lines that differ in their snow mold tolerance (SMT) or susceptibility (SMS) and freezing tolerance (FrT) or susceptibility (FrS). Two parent varieties and eight recombinant inbred lines (RILs) were analyzed. The selected RILs represent four combinations of tolerance: SMT/FrT, SMT/FrS, SMS/FrT, and SMS/FrS. It is hypothesized that carbohydrate accumulation and transcript expression will differ between sets of RILs. Liquid chromatography with a refractive index detector was used to quantify carbohydrate content at eight time points over the cold treatment period. Polysaccharide and sucrose content differed between SMT and SMS RILs at various time points, although there were no significant differences in glucose or fructose content. Glucose and fructose content differed between FrT and FrS RILs in this study, but no significant differences in polysaccharide or sucrose content. RNAseq was used to investigate differential transcript expression, followed by modular enrichment analysis, to reveal potential candidates for other mechanisms of tolerance, which included expected pathways such as oxidative stress, chitinase activity, and unexpected transcriptional pathways. These differences in carbohydrate accumulation and differential transcript expression begin to give insight into the differences of wheat lines when exposed to cold temperatures.

## 1. Introduction

Winter wheat (*Triticum aestivum* L.) plants require a period of cold temperatures to shift from vegetative growth to reproductive growth, a transition known as vernalization. Vernalization is primarily controlled by *VRN1* genes, such that the recessive alleles result in a requirement for cold temperatures to initiate reproductive growth [1]. A typical greenhouse treatment to fulfill the vernalization requirement consists of approximately 6–8 weeks of growth at 4 °C under short day-length [2,3]. Natural winter conditions can be far more stressful to a wheat plant, however. Thus, in the autumn, winter wheat undergoes cold acclimation, which is a process involving many physiological, biochemical, and genetic changes that prepare a plant to survive the potentially freezing and snow-covered winter.

During winter, young wheat plants may be exposed to freezing temperatures when not insulated with snow cover. To avoid cell death associated with freezing temperatures, winter wheat accumulates simple carbohydrates, including sucrose, glucose, and fructose, in the crown region, thus lowering the freezing point of the cell and enabling the plant to survive [4,5]. These carbohydrates also serve as an energy source for the plant while photosynthesis is diminished due to cold temperatures and potential snow cover [4,6]. When there is snow cover, a diverse complex of fungal and fungal-like pathogens, referred to as ‘snow mold’ can also take advantage of a plant’s carbohydrate stores for energy. Persistent snow cover, which insulates plants from freezing ambient temperatures, provides a favorable environment for these psychrophilic, or “cold-loving”, pathogens [7]. To avoid infection by snow mold pathogens, winter wheat accumulates fructans, which are large carbohydrates consisting primarily of chains of fructose molecules [8]. These complex carbohydrates are thought to be unusable for snow mold pathogens but can be broken down by the plant for energy as needed [6,9]. Accumulation of any type of carbohydrate, whether simple or complex, can contribute to freezing tolerance, although only complex carbohydrates have been shown to contribute to snow mold tolerance [10,11,12,13].

Mohammad et al. [14] found that winter wheat varieties with moderate to high snow mold tolerance accumulated and maintained larger reserves of nonstructural carbohydrates than varieties with low to moderate snow mold tolerance. Of the non-structural polysaccharides accumulated in wheat, fructans have been shown to account for up to 30% of the dry weight of cold-acclimated seedlings [15]. In a study comparing carbohydrate levels among cultivars varying in freezing and snow mold tolerance, Yoshida et al. [8] observed that the most freezing-tolerant variety accumulated the most mono- and di-saccharides and the least polysaccharides. In direct contrast, the most snow mold tolerant variety accumulated the most polysaccharides and the least mono- and di-saccharides, although it had the greatest reserves of both poly- and simple saccharides remaining in the spring. Accordingly, a third variety with moderate tolerance to each snow mold and freezing accumulated intermediate amounts of both polysaccharides and simple saccharides. Come spring, the remaining reserves of simple sugars were similar between the moderately tolerant variety and the snow mold tolerant variety, but the reserves of polysaccharides in the moderately tolerant variety were nearly zero, as in the freezing tolerant variety. For both freezing tolerance and snow mold infection, it has been suggested that the primary difference between tolerant and susceptible wheat varieties is the amount of carbohydrates remaining at the end of winter [16]. Whatever the role of fructans, the amount accumulated and the rate of use is likely to determine the degree of snow mold tolerance [17]. The genes responsible for fructan synthesis encode for the following enzymes: *1-SST*, *1-FFT*, *6-SFT*, and *6G-SFT* [18]. All of these genes are under the control of the transcription factor *MYB13*. The enzymes involved in fructan metabolism are *1-FEH*, *6-FEH*, and *6-KEH* [19,20].

*VRN-1* interacts with the *FR-2* locus and copy number and sequence variations at each of these loci account for much of the variation between freezing tolerant and susceptible lines [21]. In addition, copy number variation in the *CBF* (C-repeat binding factors) regulon at the *FR-2* locus contributes to snow mold tolerance [22]. The *CBF* regulon consists of numerous *COR* (cold-responsive) genes and transcription factors including the well-characterized, temperature-induced genes, *COR14b* and *Wcs120*. These genes encode a chloroplast-protective peptide to prevent photodamage and a dehydrin suspected to prevent freezing-related dehydration [23]. Additional wheat dehydrin genes, *Lea* and *Wdhn13*, have also been correlated with protecting proteins and DNA from freezing damage [24,25]. Antioxidant enzymes and antifreeze proteins have also been indicated in defense against freezing damage [26,27,28,29,30]. The numerous genes involved in freezing tolerance reflect the variety of mechanisms involved in freezing tolerance.

Similarly, snow mold tolerance is a complex trait involving carbohydrate dynamics, pathogenesis-related proteins, and lipid transfer proteins. Chitinase and glucanase were induced in cold-acclimated wheat plants, as were the antipathogenic polypeptides resulting from expression of a novel defensin-like gene, *Tad1* [4,31]. Several classes of lipid transfer proteins are also activated in response to cold temperatures and are suspected to strengthen structural barriers and arrest microbial growth [32]. No specific R genes for snow mold are known, and snow mold tolerance seems to be more of a general defense response [4].

Previous studies have verified the expression of snow mold and cold tolerance-related genes using semi-quantitative RT-PCR. *VRN1-A* in winter wheat was found to increase transcription when exposed to short daylight conditions compared to long daylight conditions [33]. *COR14b* was found to be expressed at higher levels in the cold-tolerant “maintained vegetative phase” mutant wheat [34] and for freezing conditions in the cultivar Yangmai 16 [35]. *CS120* was also found to increase in Yangmai 16 when exposed to freezing conditions [35]. *CS120* and *wdhn13* transcription levels were found to be highly correlated with freezing tolerance [25]. *Wdhn13* was found to increase transcription during drought conditions in a drought-tolerant wheat line and other members of the *Triticeae* genus [36]. *TAD1* overexpression via a transgenic maize ubiquitin promoter has been shown increase tolerance to speckled snow mold and Fusarium head blight [37].

Analyzing the patterns of carbohydrate accumulation and use among recombinant inbred lines that vary in their tolerance to freezing stress and snow mold infection is expected to elucidate the connection between carbohydrate metabolism and both stresses. Past studies have focused on differences in carbohydrate storage between unrelated winter wheat varieties. In the present study, we analyze differences among recombinant inbred lines derived from parents that differ in their tolerance to both freezing stress and snow mold infection. It is hypothesized that carbohydrate dynamics differ between the tolerant and susceptible parents. The objectives of this study were to (1) demonstrate distinct patterns of carbohydrate accumulation in accordance with their different combinations of freezing and snow mold tolerance in a recombinant inbred line population, and (2) investigate differences in transcript expression during cold hardening to detect differences between tolerant and susceptible recombinant inbred lines (RILs).

## 2. Results

### 2.1. Carbohydrate Accumulation

**Figure 1:**
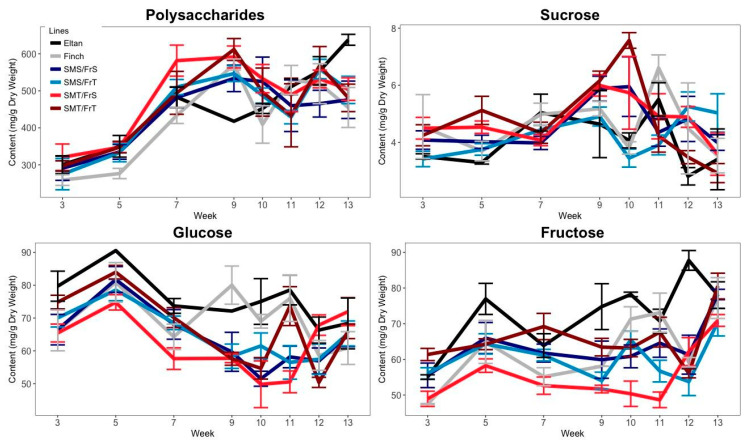
Changes in polysaccharide and simple carbohydrate content in wheat differing for snow mold and freezing tolerance after a cold treatment over 13 weeks at 4 °C.

Concentration of polysaccharides, sucrose, glucose, and fructose were compared between tolerance categories and time points to detect a relationship that could suggest a role in tolerance to snow mold and freezing. Analysis of variance of the polysaccharide content of each RIL at each time point showed no significant differences between the replicates (*p* = 0.68) or tolerance categories (*p* = 0.20), but significant differences existed between time points (*p* < 0.01) (Figure 1). Polysaccharide content increased in weeks 3 through 9 in RILs among all four tolerance categories and leveled off in later weeks. The maximum value of polysaccharide concentration was reached in week 9 amongst the RILs with both snow mold tolerance and freezing tolerance. By contrast, the minimum concentration of polysaccharides was reached in week 11 by the RILs with freezing tolerance and susceptibility to snow mold.

**Figure 2:**
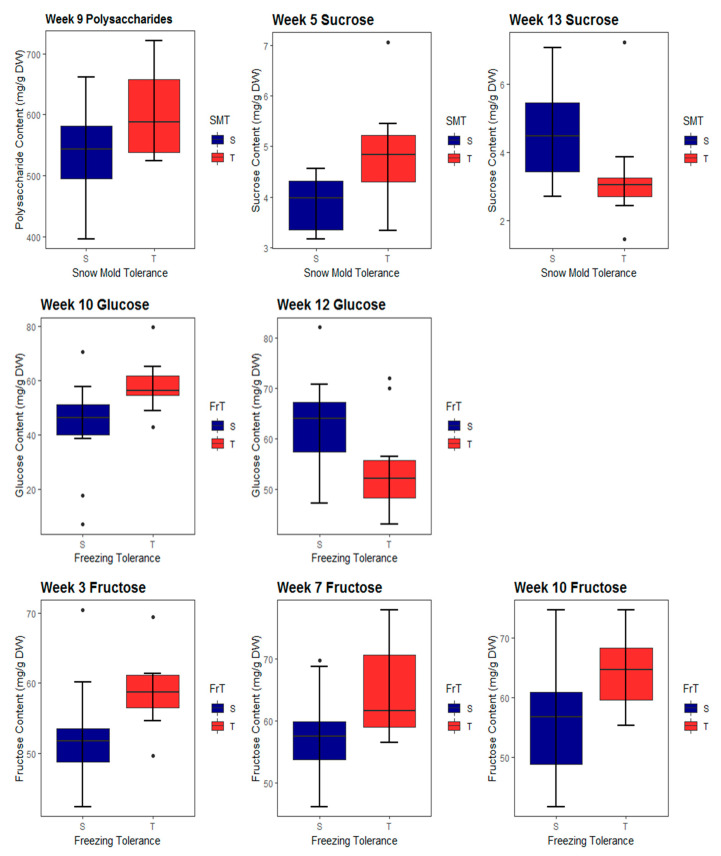
Time points at which significant differences in carbohydrate content exist between tolerant and susceptible recombinant inbred lines (RILs) of winter wheat.

Analysis of variance of each of the polysaccharide, sucrose, glucose, and fructose contents, demonstrated no significant differences among the replicates. Polysaccharide content increased from week 3 to week 9, at which time the maximum polysaccharide content was reached in RILs across all tolerance categories, although it was greatest in RILs with SMT (Figure 1). Polysaccharide content only differed significantly between SMT and SMS RILs at week 9 (*p* = 0.04; Figure 2). At no time point did polysaccharide content differ significantly between FrT and FrS RILs. Sucrose content varied little between weeks 3 and 7 and reached its maximum in weeks 9 and 10 before decreasing toward initial levels in later weeks (Figure 1). Sucrose content differed between SMT and SMS RILs at weeks 5 (*p* = 0.01) and 13 (*p* = 0.03) (Figure 2), but it never differed significantly between FrT and FrS RILs. Glucose content peaked at week 5, decreased in all tolerance categories until week 9, and varied at succeeding time points (Figure 1). It differed between FrT and FrS RILs in weeks 10 (*p* = 0.01) and 12 (*p* = 0.04; Figure 2). Fructose content was steady in weeks 3 through 12 and peaked at the final time point, when both the polysaccharide and sucrose content decreased (Figure 1). Fructose content differed between FrT and FrS RILs at weeks 3 (*p* = 0.02), 7 (*p* = 0.02), and 10 (*p* = 0.02).

### 2.2. Transcript Expression of Genes of Interest

**Figure 3:**
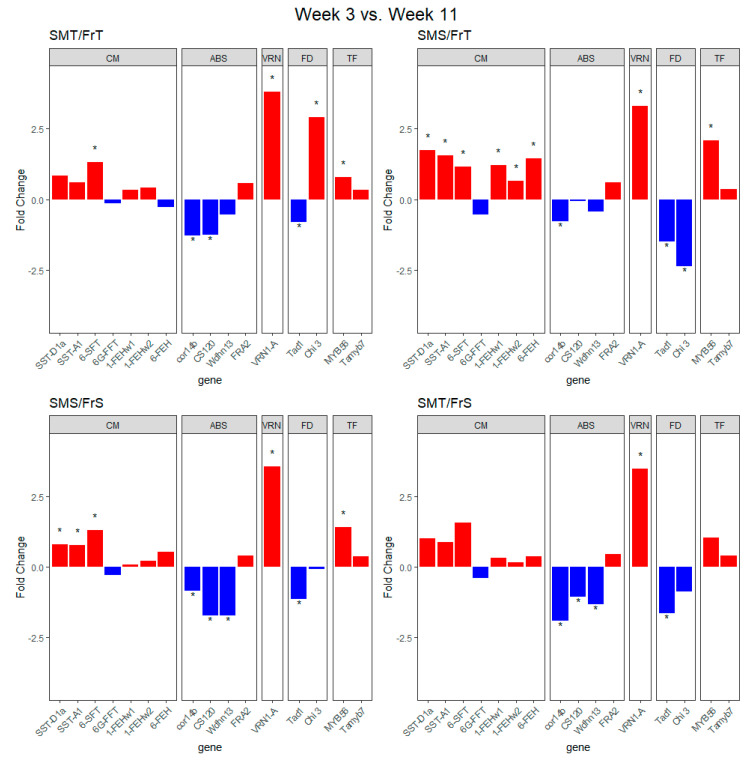
Fold change transcript expression between dates within RILs of different categories of freezing and snow mold tolerance. Genes were grouped and labeled by the hypothetical function categories; carbohydrate modification, drought/cold stress, vernalization, fungal defense, and transcription factor represented by the abbreviations CM, ABS, VRN, FD, and TF, respectively. “*” denotes genes with significant differences (q < 0.05) in transcript expression between week 3 and week 11 of the study.

Transcripts of genes of interest from previous studies described in the introduction were tested for differential expression, and significant expression differences were detected for within-genotype time point comparisons and between-genotype comparisons at both 3 and 11 weeks of vernalization. *VRN1-A* is significantly upregulated in all the RILs in week 3 compared to week 11 (Figure 3). For the SMT/FrT RIL, *6-SFT*, *Chi 3*, and *MYB56* were upregulated significantly whereas *cor14b*, *CS120*, and *Tad1* were downregulated from week 3 to week 11 during the cold treatment (Figure 3). In the SMS/FrT RIL, all of the carbohydrate-related genes were significantly upregulated whereas *cor14b* and both fungal defense genes were significantly downregulated (Figure 3). In the SMT/FrS RIL, the abiotic stress related genes, *cor14b*, *CS120*, and *Wdhn 13*, along with the fungal defense gene, *Tad1*, were significantly downregulated in week 11 compared to week 3 (Figure 3). The SMS/FrS RIL had significant up regulation of the *SST-D1a*, *SST-A1*, and *6-SFT* carbohydrate modification-related genes and *MYB56* between week 3 and week 11 of the cold treatment (Figure 3). The SMS/FrS RIL also had down regulation of *cor14b*, *CS120*, *Wdhn 13*, and *Tad1* (Figure 3) between week 3 and week 11.

**Figure 4:**
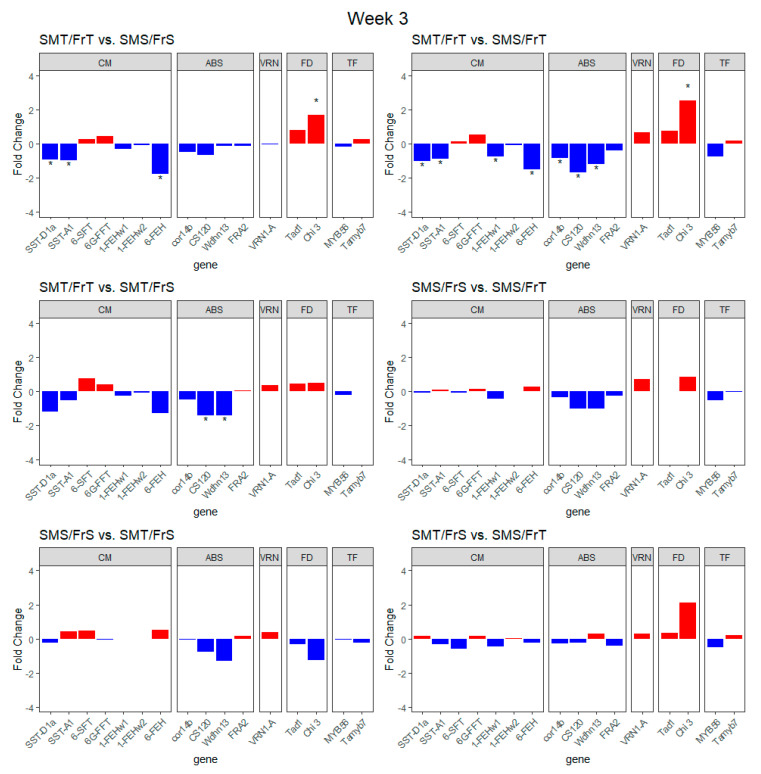
Fold change transcript expression between RILs of different classes of freezing and snow mold tolerance at week 3. Genes were grouped and labeled by the hypothetical function categories; carbohydrate modification, drought/cold stress, vernalization, fungal defense, and transcription factor represented by the abbreviations CM, ABS, VRN, FD, and TF, respectively. “*” denotes genes with significant differences (q < 0.05) in transcript expression between RILs at week 3 of the study.

Genotype comparisons of transcript expression (Figure 4) were performed at week 3 in the cold treatment and revealed that only the SMT/FrT RIL had significant differences with other RILs. Genes related to carbohydrate modification and abiotic stress response were downregulated while *Chi 3* was upregulated in the other RILs as compared to SMT/FrT. In week 3 the carbohydrate processing genes *SST-D1a*, *SST-A1*, and *6-FEH* were downregulated and the *Chi 3* gene was upregulated in the SMS/FrS RIL compared to the SMT/FrT RIL (Figure 4). After 3 weeks of cold treatment the SMS/FrT RIL had significantly lower transcript expression in *SST-D1a*, *SST-A1*, *1-FEHw1*, *6-FEH*, *cor14b*, *CS120*, and *wdhn 13*, while *Chi 3* had significantly higher transcript expression, compared to the SMT/FrT RIL (Figure 4). The SMT/FrT against SMT/FrS comparison at week 3 showed only a significantly lower level of transcript expression for the *CS120* and *wdhn 13* genes (Figure 4).

**Figure 5:**
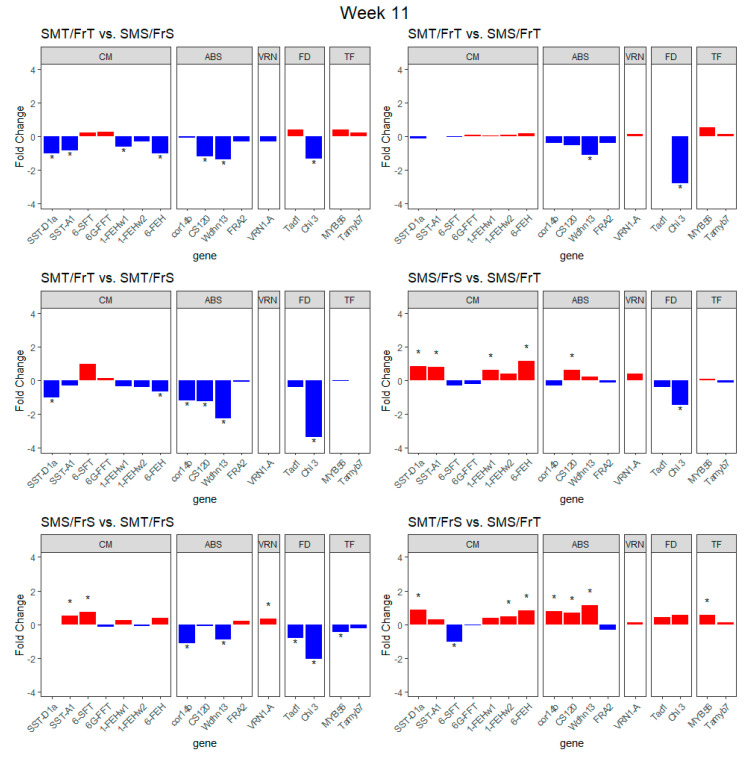
Fold change transcript expression between RILs of different classes of freezing and snow mold tolerance at week 11. Genes were grouped and labeled by the hypothetical function categories; carbohydrate modification, drought/cold stress, vernalization, fungal defense, and transcription factor represented by the abbreviations CM, ABS, VRN, FD, and TF, respectively. “*” denotes genes with significant differences (q < 0.05) in transcript expression between RILs at week 11 of the study.

Transcript expression levels of known genes were compared between genotypes after 11 weeks of cold treatment, revealing that the *Chi 3* gene, genes related to carbohydrate modification, and genes involved with cold/drought tolerance were significantly differentially regulated between RILs (Figure 5). The SMT/FrT RIL had significantly higher transcript expression of the *SST-D1a*, *SST-A1*, *1-FEHw1*, *6-FEH*, *CS120*, *Wdhn13*, and *Chi 3* genes compared to the SMS/FrS RIL. Compared to the SMS/FrT RIL, the SMT/FrT RIL had significantly higher transcript expression of *Wdhn13* and *Chi 3* genes (Figure 5). The SMT/FrT RIL had significantly higher transcript expression of *SST-D1a*, *6-FEH*, *cor14b*, *CS120*, *Wdhn13*, and *Chi 3* genes than the SMT/FrS RIL (Figure 5). After 11 weeks of cold treatment *SST-D1a*, *SST-A1*, *1-FEHw1*, *6-FEH*, and *CS120* were significantly upregulated while *Chi 3* was significantly downregulated in the SMS/FrT RIL compared to the SMS/FrS RIL. Transcript expression of *SST-A1*, *6-SFT*, and *VRN-1A* genes was significantly higher while *cor14b*, *Wdhn13*, *Tad1*, *Chi 3*, and *MYB56* were significantly lower in SMT/FrS compared to SMS/FrS (Figure 5). After 11 weeks of cold treatment *SST-D1a*, *1-FEHw2*, *6-FEH*, *cor14b*, *CS120*, *Wdhn13*, and *MYB56* were significantly upregulated, while *6-SFT* was significantly downregulated in the SMS/FrT RIL compared to the SMT/FrS RIL (Figure 5).

### 2.3. Modular Gene Enrichment

Modular enrichment analysis was used to identify other pathways contributing to tolerance. Comparisons were made between individual RILs at two different time points (Table 1) and between different RILs at each of the two time points (Table 2). The cross-time comparisons in Table 1 show that the SMS/FrS RIL had different expression levels only in the peroxisome and oxidative stress module, whereas the SMT/FrT RIL was the only RIL where the modified Fisher’s exact test for enrichment yielded non-significant enrichment in expression for gene ontology (GO) terms in that cluster. The metabolism and redox reactions and nuclear binding and protein modification modules only changed significantly in SMT RILs. The cell wall carbohydrates and chitin metabolism module had a significant enrichment score in the FrT RILs in the cross-time comparison. A beta-glucan synthesis module was only significant in the SMS/FrT RIL.

When comparing different RILs within the same time point, a module containing carbohydrate metabolism gene transcripts was significant only in the SMT/FrT and SMS/FrT comparison (Table 2). The module associated with cell wall and chitin metabolism was significant for at least one of the two time points in all comparisons between SMS and SMT except for the SMT/FrT and SMS/FrS comparison. However, the cell wall and chitin metabolism module also appeared significant for the SMS/FrS and SMS/FrT comparison. The most frequent significant module across the genotype comparisons contained genes with GO terms related to metabolism and redox reactions. The metabolism and redox module were significant in all comparisons except the SMT/FrT and SMT/FrS comparison, suggesting that response to oxidative stress is a component in both freezing and snow mold tolerance.

## 3. Discussion

The accumulation and maintenance of carbohydrates in the crown region has been shown, repeatedly, to correspond to tolerance of snow mold and freezing temperatures in winter wheat. Such studies, however, have been conducted using unrelated wheat varieties [8,16]. In this study, comparisons of carbohydrate dynamics between RILs with different combinations of tolerance suggested that carbohydrate dynamics did not vary along the divisions used to categorize the RILs. Making comparisons between SMT and SMS RILs and between FrT and FrS RILs showed significant differences for various carbohydrates at a few key time points, which varied for individual carbohydrates. Although the carbohydrate content differed significantly at several time points, the relationship between greater carbohydrate content and tolerance was inconsistent. This inconsistency may be due, in part, to the RILs chosen to represent the four tolerance categories, particularly for those RILs that were tolerant to only one of the traits. Because of the shared pathways involved in these tolerance traits, it is difficult to find plants that are fully tolerant to snow mold while also fully susceptible to freezing. Nevertheless, the tolerance scores of the tolerant RILs were significantly greater than those of the susceptible RILs, indicating that the categories were sufficiently distinct to potentially reveal the differences responsible for the variation in their tolerance levels. In addition, tolerance is likely conferred by traits other than carbohydrate accumulation, so the transcript expression of known genes related to snow mold and freezing tolerance was investigated, and modular enrichment analysis was performed to investigate potential mechanisms.

Modular enrichment analysis revealed expected mechanisms of tolerance to freezing and snow mold. However, modular enrichment analysis is limited by unidirectional results and was not able to entirely separate the mechanisms for freezing and snow mold tolerance due to the complexity of the underlying pathways and complications of trying to analyze the traits independently. For example, the appearance of a chitinase and cell wall metabolism cluster made sense where snow mold tolerance contrasted. However, the cell wall and chitin metabolism module were also significant for the SMS/FrS and SMS/FrT comparison suggesting that freezing tolerance is improved in lines with increased transcript expression of genes related to fungal defense, perhaps due to linkage between these genes and others contributing to freezing tolerance. The significant enrichment of metabolism and redox reaction pathways in most comparisons supports other results indicating redox responses in tolerance to abiotic stress [38,39,40] as well as a defense to fungal infections [41,42,43]. However, because modular enrichment uses the mean of values generated from unidirectional tests, it is not clear what lines expressed more or less transcripts for a given GO term or enrichment module. The carbohydrate metabolism module was significant only in SMT/FrT and SMS/FrT comparison after cold treatment, consistent with the hypothesis of differing carbohydrates for snow mold tolerance and increased fructan accumulation. Differences in carbohydrate metabolism were significant only after cold treatment, likely because the plants respond differently to changes in the environment.

To parse out the modular enrichment analysis further, differential transcript expression analysis of specific genes related to snow mold and freezing tolerance was performed. Comparing each RIL’s transcript expression at weeks 3 and 11 reveals three genes that are differentially expressed regardless of the tolerance category: *VRN1-A*, *cor14b*, and *Tad1*. The upregulation of *VRN1-A* is consistent with the expected vernalization response to the short day and cold temperature treatment. The down-regulation of *cor14b* and *Tad1*, both known to be cold-induced, likely reflects the decline in cold acclimation associated with the fulfillment of the vernalization requirement. *Chi 3* was significantly upregulated in week 11 compared to week 3 for the SMT/FrT RIL, while being significantly downregulated in the SMS/FrT, suggesting *Chi 3* has a role in snow mold tolerance. In the genotype comparisons, *Chi 3* is upregulated in week 3 and down regulated in week 11 for SMT RIL compared to SMS RIL, suggesting that upregulation of chitinase producing genes early in the vernalization process contributes to tolerance to snow mold. The results of the *Chi 3* in the cross-time comparisons showed that the cell wall carbohydrate and chitin metabolism module is likely upregulated in SMT/FrT while being downregulated in the SMS/FrT. This suggests that higher chitinase levels at week 11 compared to week 3 of vernalization play a part in tolerance to snow mold.

In winter rye, chitinase enzymes *cor14b*, *CS120* and *Wdhn13* were downregulated while *FRA2* was upregulated during vernalization, suggesting *cor14b*, *CS120*, and *Wdhn13* are activated by cold acclimation pathways [9]. In this study, at both 3 and 11 weeks of cold treatment the *cor14b*, *CS120*, and *Wdhn13* genes tended to be more highly expressed in FrT RIL compared to FrS RIL, suggesting they are important to freezing tolerance. The similar profile of results in the freezing/drought tolerance genes suggest those genes are triggered by vernalization across RIL, although comparisons of genotypes at those times show differences of the levels between RIL of differing snow mold and cold tolerance.

Although our experiment measuring carbohydrate accumulation in the crown tissue was unable to identify conclusive differences between lines, the differentially expressed genes (DEGs) from the RNA-seq corroborate the results of Mohammad et al. [14]. In both week 3 and 11, the FrS RIL had significantly less transcript expression of the fructan synthesis genes *SST-D1a* and *SST-A1*, suggesting that fructan accumulation is related to cold tolerance, corroborating the results of Pollock and Jones [15]. At week 11 the SMT/FrT and SMS/FrT had significantly differentially expressed DEGs associated with drought/cold tolerance and *Chi 3*, but all of the carbohydrate modification/synthesis related genes had almost no difference between RIL, providing further evidence that non-structural carbohydrate accumulation is involved in freezing tolerance.

Sanderson et al. [44] examined genes regulated by a transcription factor involved in cold tolerance and found significant regulation differences in genes with GO terms relating to response to oxidative stress, response to fungal stress, and DNA binding. Sanderson et al. [44] also found dehydrin genes activated during cold acclimation. These results corroborate some of the pathways we found; however, molecular pathways such as metabolism, cellulose synthesis, and membrane transport/ion transport should be examined further to validate or understand the role of these pathways in freezing and snow mold tolerance in plants. The output of the modular enrichment analyses (Supplemental Materials, File 2) contains the differentially expressed genes associated with each FUNC-E cluster providing researchers with potential genes to characterize.

In conclusion, the hypothesis that patterns of carbohydrate accumulation would reflect patterns of tolerance, over time or at key time points, was not supported by the results of this study. This suggests that the observable differences in tolerance to snow mold and freezing in this population were not a direct result of differences in carbohydrate dynamics due to differences in other pathways contributing to these highly quantitative tolerance traits, or the carbohydrate experiment was not specific enough to detect differences. RNA-Seq was able to detect genes associated with fructan synthesis, even though the results of the fructan carbohydrate experiment showed little significant differences. RNA-Seq analysis was also able to detect many other genes and gene families (such as chitinase) that were up- or downregulated between tolerant and susceptible RIL. Further studies could examine shared pathways to determine how they contribute to each freezing and snow mold tolerance individually. Developing isogenic lines that represent all four tolerance combinations may also be useful, although challenging given the shared pathways that contribute to both phenotypes, instead of single genes controlling tolerance.

## 4. Materials and Methods

### 4.1. Plant Material and Growing Conditions

Eight recombinant inbred lines (RILs) were selected from a RIL population derived from a cross between soft white winter wheat cultivars, “Finch” (PI628640) [45] and “Eltan” (PI5369940) [46]. Finch and Eltan differ in their tolerance to snow mold and freezing stress, and segregation for these traits was observed among the RILs [47]. The eight lines chosen for this experiment represent four categories of snow mold tolerance (SMT) or susceptibility (SMS) and freezing tolerance (FrT) or susceptibility (FrS): SMT/FrT, SMT/FrS, SMS/FrT, SMS/FrS, based on preliminary data.

For the carbohydrate quantification experiment, the eight RILs and two parents were included in three randomized complete blocks in three trials. They were planted in seedling starter trays (East Jordan Plastics, Inc., East Jordan, MI, USA, A 8-06) and allowed to germinate and grow to the 3-leaf stage at 22 °C with 16 h day-length. Plants were then transferred to the cold treatment conditions, 4 ^o^C with 12 h day-length. Samples were collected at eight time points as the 2 cm section of crown tissue directly above the roots [8,48]. The collections occurred at weeks 3, 5, 7, 9, 10, 11, 12, and 13 after treatment. For the transcript expression profile experiment, four RILs representing the four tolerance categories (Table 3) and the two parents were included in three randomized complete blocks in one trial that was grown under the same conditions for the same period of time as described previously. Samples were collected at weeks 3 and 11 as 1 cm (sample size based on expected yield of extracted total RNA) of crown tissue taken from the base of the crown, and pooled across five individual plants. All samples were immediately frozen in liquid nitrogen and stored at −80 °C until further processed.

### 4.2. Carbohydrate Extraction and Quantification

Carbohydrate extraction was carried out via a protocol slightly modified from Ekvall [49]. Samples were lyophilized, ground with steel beads using a Geno/Grinder 2010, and then weighed into 10 mg samples. To each 10 mg tissue sample, 2.0 mL of 50% ethanol was added. Samples were mixed via vortex and centrifuged, so the supernatant could be removed and retained. An additional 2.0 mL of 50% v v^−1^ ethanol was added to the first tube, mixed via vortex, and then shaken for 2 hrs at room temperature. Samples were centrifuged and the supernatant retained and pooled with previous for a total volume of approximately 4.0 mL. A 2.0 mL aliquot was taken from the pooled supernatant and added to 1.0 mL 90% v v^−1^ ethanol. The samples were then dried in a hot water bath at 60 °C with forced air over the top of each vial. Once re-eluted with 1.0 mL 18.0 mΩ H_2_O, the samples were shaken and filtered through micro syringe filters (PES 13 mm 0.45 μM) into vials. Samples were analyzed in an Agilent 1100 series HPLC (Agilent Technologies, Santa Clara, CA, USA) with an autosampler that injected 60 μL into the HPLC column, for separation using the Shodex KS 802 and Shodex KS 803 columns (Showa Denko K.K., Tokyo, Japan) in tandem [8]. Sucrose, fructose, and glucose were quantified using external standards. For polysaccharides, peak area comprising polysaccharide content (dp ≥ 3) was summed and compared to a standard solution of inulin (Sigma Chemical Co., St. Louis, MO, USA).

### 4.3. Statistical Analysis

Polysaccharide, sucrose, glucose, and fructose content were compared between SMT and SMS lines and between FrS and FrT lines at each time point using the lme function in the nlme package in R [50].

### 4.4. RNA Extraction, RNAseq, and Modular Enrichment Analysis

FERIL-20, FERIL-74, FERIL-94, and FERIL-128 were used with three biological replicates containing 5 crown tissue samples from each plant at each time point for RNA-seq analysis. Validation with qPCR was not performed because a number of studies have shown that using RNA-seq or qPCR will yield correlated results and the same conclusions [51,52,53,54]. Samples were ground with a mortar and pestle using liquid nitrogen to maintain freezing temperatures during grinding. RNA was extracted using the GeneJET Plant RNA Purification Mini Kit, following the associated protocol (Thermo Scientific). An aliquot of extracted RNA was sent to the Washington State University-Spokane Genomics Core Lab for library preparation, RNA-seq, and data analysis. Library construction and cDNA sequencing were performed on an Illumina platform. Reads were aligned to the *T. aestivum* reference genome (IWGSC RefSeq v1.0) using Bowtie2 [55] software. Differential transcript expression was estimated using Cufflinks [56]. Differentially expressed genes (DEGs) were identified as significant when the genes had a q-value from Cufflinks below 0.05 (Supplemental Materials, File 1). *VRN1-A*, *cor14b*, *CS120*, *Wdhn13*, *Tad1*, *SST-D1a*, *SST-A1*, *6-SFT*, *6-GFFT*, *1-FEHw1*, *1-FEHw2*, *6-FEH*, *MYB56*, *Tamyb7* genes and the *Chi3* protein gene were examined separately from the modular enrichment analysis to develop a profile of the transcript expression of known and hypothetical genes. The genes tested separately were determined to be significant if the q-value was below 0.05. Gene ontology (GO) terms were extracted from the iwgsc_refseqv1.0 FunctionalAnnotation_v1__HCgenes_v1.0 gff3 file and the definitions for the terms were obtained by querying the GO database using the GO.db library [57] in R statistical programming language (Supplemental Materials, File 4). Gene set analysis and modular enrichment analysis were investigated using FUNC-E (Supplemental Materials, File 3) with default parameters [58]. FUNC-E performs the same methods for modular enrichment analysis as DAVID [59] but allows users to enter annotation for species not supported by DAVID. FUNC-E calculates a *p*-value for enrichment of each GO term present using a modified fisher’s exact test, then terms with significant kappa coefficients are clustered together and the negative log geometric mean of the *p*-values for terms in each cluster is reported as the enrichment score. Enrichment scores above 1 are considered significant when α = 0.01.

## Figures and Tables

**Table 1:** Group enrichment scores for modules differentially regulated for cross-time comparisons within the same RIL. (*p*-value significant at α = 0.05).

Module	SMT/FrT	SMS/FrS	SMT/FrS	SMS/FrT
Peroxizome and oxidative stress		2.2889	2.6257	6.6852
Metabolism and redox reactions	5.6142		4.2229	
Nuclear binding and protein modification	6.2492		4.8104	
Cell wall carbohydrates and chitin metabolism	2.6723			9.4938
Beta-glucan synthesis			2.0558	

**Table 2:** Group enrichment score for putative modules related to molecular pathways differentially regulated between wheat RILs with differing levels of snow mold and freezing tolerance at the same time point. (*p*-value significant at α = 0.05).

Comparisons	SMT/FrT and SMS/FrS		SMT/FrT and SMT/FrS		SMT/FrT and SMS/FrT		SMS/FrS and SMT/FrS		SMS/FrS and SMS/FrT		SMT/FrS and SMS/FrT	
Module Week:	3	11	3	11	3	11	3	11	3	11	3	11
Acyl transferase activity	3.44											
Carbohydrate metabolism		2.62										
Cell wall carbohydrates and chitin metabolism					8.38	3.36		4.35	7.78		6.60	
Cellulose synthesis	5.75											
Membrane transport and ion channels	2.99											
Metabolism and redox reactions	3.42	2.69			4.35			3.09	2.99		6.81	
mRna modification	2.69											
Nuclear binding and protein modification			9.16	5.87	37.67							2.88
Peroxisome and oxidative stress		5.21						4.57				

**Table 3:** Data (raw scores) used to determine representatives of the tolerance categories in comparison to the tolerant and susceptible parents. Bold RILs were used in the transcript expression experiment.

Category	RIL	SMT Score ^a^	FrT % ^b^
tolerant parent	Eltan	6.33	68
SMT/FrT	**FERIL-20**	7	81
	FERIL-71	6.67	85
SMT/FrS	**FERIL-94**	6	15
	FERIL-103	6	8
SMS/FrT	**FERIL-128**	3.33	58
	FERIL-132	3	68
SMS/FrS	**FERIL-74**	1.67	5
	FERIL-101	2	8
susceptible parent	Finch	4.33	1

^a^ Snow mold tolerance was evaluated on a scale of 0 (severe susceptibility) to 10 (strong tolerance). ^b^ Freezing tolerance was recorded as percent survival, thus higher numbers indicate greater tolerance.

## Supplementary Materials

The following are available online at https://www.mdpi.com/2223-7747/9/11/1416/s1. Supplemental File 1. Input RNA-Seq expression data of RIL varying for their tolerance/susceptibility to freezing temperatures and snow mold. Supplemental File 2. Output file of the FUNC-E analysis of RIL varying for their tolerance/susceptibility to freezing temperatures and snow mold. Supplemental File 3. Input file of the FUNC-E analysis of RIL varying for their tolerance/susceptibility to freezing temperatures and snow mold. Supplemental File 4. R code used to analyze RNA-Seq expression data of RIL varying for their tolerance/susceptibility to freezing temperatures and snow mold.

## Abbreviations

RIL	recombinant inbred line
GO	gene ontology
BLUPs	best linear unbiased predictions
FrT	freezing tolerant
FrS	freezing susceptible
SMT	snow mold tolerant
SMS	snow mold susceptible
