# Supplementary material for: Carbohydrate Accumulation and Differential Transcript Expression in Winter Wheat Lines with Different Levels of Snow Mold and Freezing Tolerance after Cold Treatment
Source: Plants (Basel). 2020 Oct 23;9(11):1416. doi: 10.3390/plants9111416 (PMC7690702; doi:10.3390/plants9111416)
Supplement: Supplementary file 1 [file plants-09-01416-s001.zip › supplementalFiles/expressionData/originalData/20190415_RNA_Seq_Method_for_Kruse.docx]

**RNA Sequencing and Data Analysis**

The integrity of total RNA was assessed using Fragment Analyzer (Advanced Analytical Technologies, Ankeny, IA) with the High Sensitivity RNA Analysis Kit. RNA Quality Numbers (RQNs) from 1 to 10 were assigned to each sample to indicate its integrity or quality. “10” stands for a perfect RNA sample without any degradation, whereas “1” marks a completely degraded sample. RNA samples with RQNs ranging from 8 to 10 were used for RNA library preparation with the TruSeq Stranded mRNA Library Prep Kit (Illumina, San Diego, CA). Briefly, mRNA was isolated from 2.5 µg of total RNA using poly-T oligo attached to magnetic beads and then subjected to fragmentation, followed by cDNA synthesis, dA-tailing, adaptor ligation and PCR enrichment. The sizes of RNA libraries were assessed by Fragment Analyzer with the High Sensitivity NGS Fragment Analysis Kit. The concentrations of RNA libraries were measured by the StepOnePlus Real-Time PCR System (ThermoFisher Scientific, San Jose, CA) with the KAPA Library Quantification Kit (Kapabiosystems, Wilmington, MA). The libraries were diluted to 2 nM with RSB (10 mM Tris-HCl, pH8.5) and denatured with 0.1 N NaOH. Eighteen pM libraries were clustered in a high-output flow cell using the HiSeq Cluster Kit v4 on a cBot (Illumina). After cluster generation, the flow cell was loaded onto HiSeq 2500 for sequencing using the HiSeq SBS kit v4 (Illumina). DNA was sequenced from both ends (paired-end) with a read length of 100 bp. The raw BCL files were converted to FASTQ files using software program bcl2fastq2.17.1.14. Adaptors were trimmed from the FASTQ files during the conversion. On average, 40 million reads were generated for each sample.

RNA-seq data (FASTQ files) were aligned to Triticum aestivum reference genome (IWGSC RefSeq v1.0, Ensembl) using Bowtie2 (Langmead and Salzberg 2012 Nature Methods 9, 357-359)). Gene expression quantification and differential expression were analyzed using Cufflinks (Trapnell et al. 2010 Nature Biotechnology 28, 511-515). Differential gene expression was considered significant at q-value <0.05.
