# Supplementary material for: Carbohydrate Accumulation and Differential Transcript Expression in Winter Wheat Lines with Different Levels of Snow Mold and Freezing Tolerance after Cold Treatment
Source: Plants (Basel). 2020 Oct 23;9(11):1416. doi: 10.3390/plants9111416 (PMC7690702; doi:10.3390/plants9111416)
Supplement: Supplementary file 1 [file plants-09-01416-s001.zip › supplementalFiles/expressionData/originalData/DEG Trimming.docx]

An RPKM (Reads per Kilobase per Million reads) cutoff value of 0.1 was set to declare a locus expressed.

**Zouari et al. BMC Genomics 2014, 15:221**

The highly significant enriched terms were chosen by [agriGO] default P-value and false discovery rate.

**Jiang et al. 2016 Combining meta-QTL with RNA-seq data to identify candidate**

**genes of kernel row number trait in maize**

DEGs were determined with as adjusted p value threshold of ≤ 0.05 and log2 expression fold change of ≥ 1 or ≤ − 1 or ‘inf’ (where the FPKM value in one condition is zero and the other is not). Genes responsive to FCR infection were identified by two pairwise comparisons between treatments. The responsive genes after infection compared with mock were identified following the same method as DEGs: threshold of FDR ≤ 0.05 and the absolute value of log2 fold change ≥ 2 or ≤ − 2 or ‘inf’.

**Habib et al. 2017 A multiple near isogenic line (multi‑NIL) RNA‑seq approach to identify**

**candidate genes underpinning QTL**

The differentially expressed genes were considered to be significant at p-value < 0.05 and absolute fold-change ≥ 2-fold. Gene ontology analysis was performed by Blast2go software

**Camilios-Neto, et al (2014). Dual RNA-seq transcriptional analysis of wheat roots colonized by Azospirillum brasilense reveals up-regulation of nutrient acquisition and cell cycle genes.**

Differentially expressed genes were considered significant at log_e_of fold change of 1.5 and FDR level of 0.05 using the Benjamini-Hochberg method

**Hübner, et al.. (2015). RNA-Seq analysis identifies genes associated with differential reproductive success under drought-stress in accessions of wild barley Hordeum spontaneum**

To analyse the GO enrichment analysis, firstly, significantly differential expressed genes were retrieved by cutting off based on P and FDR values <0.05, respectively, and log2 fold change >1.5.

**Jeong, et al. (2017). Phosphorus remobilization from rice flag leaves during grain filling: an RNA‐seq study**
